# Myopia in the Diagnosis of Marfan Syndrome: An Important Early Sign of a Systemic Condition

**DOI:** 10.7759/cureus.23651

**Published:** 2022-03-30

**Authors:** Bhavini B Prajapati, Jennifer Monti

**Affiliations:** 1 Internal Medicine - Pediatrics, Maine Medical Center, Portland, USA; 2 Cardiology, Maine Medical Center, Portland, USA

**Keywords:** fbn1 mutation, ectopia lentis, variant of unknown significance, aortic dissection, marfan syndrome

## Abstract

Marfan syndrome is a genetic connective tissue disorder that is a frequent cause of aortic dissection in younger patients. We report a case of a patient with a history of ectopia lentis in his third decade and abdominal striae since adolescence, who presented with Stanford type A dissection at age 48.

## Introduction

Marfan syndrome is a connective tissue disorder with variable clinical presentation. Ectopia lentis and aortic dilation are core features reflected in the revised Ghent criteria for diagnosing this condition [1]. Aortic dissection in these patients can be quite extensive and early screening for aortic dilation can help reduce the associated morbidity and mortality [2]. Genetic screening provides the opportunity to identify individuals and families at risk, but actionable results are limited by the current data available on genotype-phenotype associations. The presence of novel mutations, particularly those labeled variants of unknown significance (VUS), in patients diagnosed with Marfan syndrome permit the attribution of clinical significance.

## Case presentation

A 48-year-old male presented to the emergency department with altered mental status and progressive back pain. His wife reported that his symptoms started two hours before his presentation and prior to that he had been in his normal state of health. Vital signs revealed a blood pressure of 84/44 mmHg and a heart rate of 56 beats per minute. Physical exam showed a disoriented male with notable striae across his back and chest.

The patient was a lifelong non-smoker who had a history of hyperlipidemia and obesity. He was not taking any medications. His surgical history was significant for a cholecystectomy, gastric sleeve, bilateral inguinal hernia repairs, and remote lens replacement surgery for bilateral ectopia lentis. He had initially presented 7 years prior to this current presentation to ophthalmology with first decreased visual acuity in the left followed by his right eye. He had no prior cardiac history or imaging.

**Figure 1:**
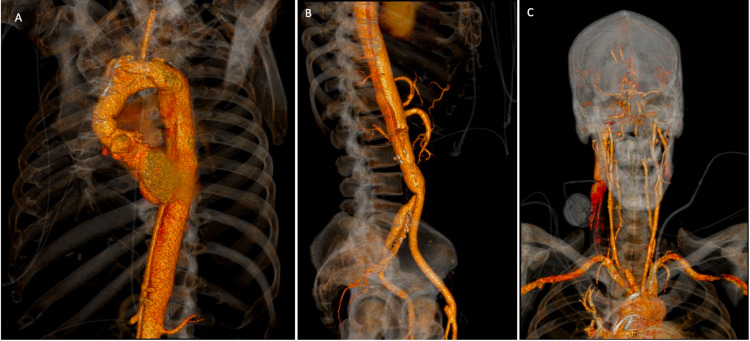
Three-dimensional computed tomography angiographic reconstruction showing extensive aortic dissection of the (A) ascending, descending, and (B) abdominal aorta. Panel C shows the extension of the dissection into the brachiocephalic artery and proximal aspects of the left common carotid artery and left subclavian artery.

A CT scan of his chest, abdomen, and pelvis was obtained revealing an extensive Stanford type A/DeBakey type 1 aortic dissection starting just distal to the aortic valve extending into the aortic arch, descending aorta, entire abdominal aorta into the bilateral iliac arteries (Figure 1A, 1B). The dissection flap was noted to extend into the brachiocephalic artery, left carotid and subclavian arteries, celiac trunk and superior mesenteric arteries.

In the setting of his altered mental status, a head CT was also obtained and showed an acute to subacute infarction within the right anterior middle cerebral artery (MCA) distribution without evidence of intracranial hemorrhage. A CT angiogram of the head and neck revealed a long segment dissection involving the right common carotid artery extending into a nearly completely occluded proximal right internal carotid artery and occlusions involving the anterior division of the right MCA bifurcation, which corresponded to the areas of infarct (Figure 1C).

The patient was taken to the operating room where he underwent a hemi aortic arch and root replacement with a Bentall procedure using a 27 mm Medtronic Freestyle bioprosthesis (Medtronic, Dublin, Ireland). Post-operatively he developed severe bradycardia resulting in cardiac arrest requiring a temporary pacemaker. Later in his course, he developed atrial fibrillation with a rapid ventricular response and mildly reduced left ventricular ejection fraction. He had a prolonged hospitalization complicated by a right middle cerebral artery stroke, left upper extremity deep vein thrombosis, and a pleural effusion requiring video-assisted thoracoscopic surgery (VATS) procedure. He was discharged to inpatient rehab and ultimately recovered.

Given the young age of presentation and history of ectopia lentis, the patient was referred to medical genetics. Detailed family history revealed a history of aortic dissection in the maternal grandmother and sudden death in several family members. The systemic score based on Ghent criteria was 4 due to the presence of myopia, pectus excavatum, and skin striae. A 37-gene panel for thoracic aortic aneurysm and dissection was obtained through Invitae Corp. (San Francisco, USA) and revealed a heterozygous mutation in the FBN1 gene (c.5150-5152; p.Lys1717del) that was classified as a variant of unknown significance. Additional family members were not offered genetic testing in this setting. 

## Discussion

Marfan syndrome is an autosomal dominant disorder of the connective tissue that affects the musculoskeletal, ocular, and cardiovascular systems. De novo mutations account for 25% of cases [3]. The majority of patients with this condition have a mutation in FBN1, which encodes the protein fibrillin-1 [4]. Fibrillins are large glycoproteins secreted in the extracellular matrix as microfibrils. They play an important role in the structural integrity and elasticity of connective tissues [3]. Fibrillins also regulate transforming growth factor-b (TGF-b) and mutations in its receptor, TGFBR2, have been found in a small number of individuals with Marfan syndrome [4]. 

Mutations reported in the FBN1 gene have a wide phenotypic range. In addition to Marfan syndrome, FBN1 mutations contribute to a range of syndromes including isolated thoracic aortic aneurysm and aortic dissection, isolated ectopia lentis, stiff skin syndrome, Weill-Marchesani syndrome 2, geleophysic dysplasia 2, acromicric dysplasia, and Marfan lipodystrophy syndrome [3]. It may be difficult to differentiate syndromes by clinical manifestations alone as distinguishing features may not develop until later in the disease course. 

Despite the vast number of mutations identified in the FBN1 gene, many have been labeled variants of unknown significance due to the lack of reported clinical associations. No cases have been reported with the specific FBN1 mutation this patient had. This variant results in the in-frame deletion of a single amino acid in exon 42, which encodes one of the seven 8-cysteine domains that are essential to the structure and function of the fibrillin proteins [3]. In the absence of previously reported clinical associations with this variant, its significance was reported as uncertain. In silico analyses have shown the deleterious effect of this mutation, but no in vivo studies have been published [5]. The presentation of this patient with classic findings of Marfan syndrome lends support to the pathogenicity of this mutation.

The diagnosis of Marfan syndrome is made based on the revised Ghent criteria, which includes positive family history, aortic root dilation (Z score ≥2), ectopia lentis, pathogenic FBN1 mutation, and the systemic score (≥7) [1]. Ectopia lentis is a specific finding for Marfan syndrome and has a high predictive value [6]. Guidelines suggest that only one additional criterion needs to be fulfilled to confirm a diagnosis of Marfan syndrome. 

This patient had ectopic lentis diagnosed years before his presentation with acute aortic dissection. Given his family history, a diagnosis of Marfan syndrome could have been made well before his dissection. Appropriate echocardiographic imaging to survey the aorta may have allowed for surgical intervention before acute dissection. 

Aortic dissection is a major cause of morbidity and mortality in Marfan syndrome. Early recognition of predisposing conditions for aortic dissection allows for preventative and staged interventions. The age of presentation of aortic disease and the rapidity with which it progresses in patients with Marfan syndrome is variable and consistent with the mutational heterogeneity [7]. Screening for aortic dilation is recommended at the time of initial diagnosis and 6 months later to assess stability followed by yearly evaluations with the goal of identifying patients who would benefit from prophylactic aortic root repair per the 2010 American College of Cardiology Foundation/American Heart Association guidelines [2]. Medical management, which consists of beta blockers and angiotensin receptor inhibitors, slows the progression of aortic dilation but does not preclude the need for surgical intervention [8,9].

In follow-up, this patient had a repeat echocardiogram that showed an ejection fraction of 30-35%. He was started on a goal-directed medical therapy and was serially monitored by cardiology, cardiothoracic surgery, vascular surgery, and the medical genetics team.

## Conclusions

This case highlights the importance of broadening the differential diagnosis in a patient who presents with a discrete problem (ectopia lentis) with systemic implications. Recognition of this syndrome has implications for the patient and potentially profound effects on family members. Future studies are needed to bolster the genotype-phenotype link of novel mutations in the FBN1 gene.
